# Bone mesenchymal stem cells transplantation combined with mild hypothermia improves the prognosis of cerebral ischemia in rats

**DOI:** 10.1371/journal.pone.0197405

**Published:** 2018-08-01

**Authors:** Min Bi, Jiawei Wang, Yidan Zhang, Longzhu Li, Linhui Wang, Ran Yao, Shijie Duan, Suijun Tong, Jianpeng Li

**Affiliations:** 1 Department of Neurology, The First Affiliated Hospital of Xiamen University, Xiamen, Fujian, China; 2 The First Clinical Medical College of Fujian Medical University, Fuzhou, Fujian, China; 3 Medical College of Xiamen University, Xiamen, Fujian, China; 4 Department of Neurology, The 184th Hospital of People’s Liberation Army of China, Yingtan, Jiangxi, China; University of South Florida, UNITED STATES

## Abstract

Bone marrow mesenchymal stem cells (BMSCs) are used as a great promising choice for the treatment of cerebral ischemia. Herein, we discuss the neuroprotective effects of the combination of BMSCs transplantation and mild hypothermia (MH) in an ischemia-reperfusion rat model. First, BMSCs were isolated using density gradient centrifugation and the adherent screening method, followed by culture, identification and labeling with DAPI. Second, adult male SD rats were divided into 5 groups: sham group (surgery without blockage of middle cerebral artery), model group (middle cerebral artery occlusion (MCAO) was established 2h prior to reperfusion), BMSCs group (injection of BMSCs via the lateral ventricle 24h after MCAO), MH group (mild hypothermia for 3h immediately after MCAO) and combination therapy group (combination of BMSCs and MH). Finally, the modified neurological severity score (mNSS) test was performed to assess behavioral function at different time points (before MCAO, before transplantation, at day 1, day 5 and day 10 after transplantation). After that, the brain was subjected to TTC staining, and the homing and angiogenesis were evaluated by immumofluorescence and immunohistochemistry. Immunofluorescence staining and Western Blot analysis were performed to calculate the percentage of the infarct area and explore glial fibrillary acidic protein (GFAP) and vascular endothelial growth factor (VEGF). Our results showed that the combination therapy significantly decreased mNSS scores (P<0.01) and reduced the percentage of the infarct area (P<0.01) than a single treatment. Moreover, the expression of GFAP and VEGF increased significantly in the combination therapy group (at day 5, day 10 after transplantation; at all time points after transplantation, respectively) compared to the single treatment groups. Taken together, it was suggested that the combination of BMSCs transplantation and MH can significantly reduce the percentage of the infarct area and improve functional recovery by promoting homing and angiogenesis, which may be a beneficial treatment for cerebral ischemia.

## 1. Introduction

Ischemia stroke is caused by cerebral artery blockage and has some severe consequences, such as impairments of motor, sensory or cognitive function[1]. Unfortunately, to date, there are no effective methods to promote the recovery of patients. Recombinant tissue plasminogen activator (rt-PA), an intravascular administered therapy, is the only proven reperfusion therapy for acute ischemia stroke at present. However, its clinical efficacy is deeply limited by the short time window[2]. Furthermore, one complication of thrombolysis is intracranial hemorrhage (ICH), which may be a serious adverse complication after thrombolytic therapy and increase the mortality and disability[3]. Therefore, it is important to seek a novel treatment for stroke.

BMSCs (bone marrow mesenchymal stem cells) have the capacity of self-renewing and differentiating into multiple cell lineages, such as bone and muscle cells, when they reside in an appropriate microenvironment [4]. At present, numerous studies have shown that BMSCs transplantation could foster the recovery of neurological function and reduce infarct size in rats with acute ischemia stroke [5–8]. The capacity of MSCs (mesenchymal stem cells) releasing growth and trophic factors or stimulating their release from endogenous cells has been suggested to make a difference in cerebral ischemia[9].

For the past decades, various studies throughout the world have shown that therapeutic mild hypothermia (MH) has neuroprotective effects and improves neurological functional outcomes in various models of brain and spinal cord injury[10–12]. Therapeutic mild hypothermia for acute brain injury can reduce tissue damage in CNS (central nervous system) and promote neurological recovery by lowering intracranial temperature. Schwab et al. [13] found that systemic mild hypothermia treatment in patients with a large area of cerebral infarction can reduce brain edema, and improve clinical prognosis. Clark et al. [12] also reported that hypothermia might reduce the neurological deficit symptoms after focal cerebral ischemia in rats, lessen the infarct volume, and promote post-injury repair.

Therefore, we intended to ascertain whether the combination of the two different neuroprotective strategies may work better. The aim of this study was to investigate the neuroprotective effects of BMSCs transplantation combined with mild hypothermia after cerebral ischemia in rats and provide theoretical basis for clinical application.

## 2. Material and methods

### 2.1. Animals

Healthy adult male Sprague-Dawley (SD) rats (aged 8–10 weeks, 250-320g) of specific-pathogen-free level (SPF) were used to establish MCAO models. Five two-week old SD rats were used for extraction of BMSCs. All SD rats were purchased from Shanghai SLAC Laboratory Animal Co., Ltd. (Shanghai, China). The study protocol was approved by the ethics committee of the First Affiliated Hospital of Xiamen University (approval No.: KY2014-016). All rats were housed in plastic cages with soft bedding under 12h light–dark cycles with free access to food and water.

#### Experimental design

The rats were then randomly divided into five groups (n = 36 rats per group) as follows:

1. Sham group: rats underwent surgery without blockage of middle cerebral artery.
2. MCAO (middle cerebral artery occlusion) group: rats underwent MCAO surgery, and were injected with PBS;
3. MH (mild hypothermia) group: MCAO rats with injection of PBS underwent mild hypothermia for 3h immediately after MCAO;
4. BMSC group: rats underwent MCAO surgery, and were injected with BMSCs via the lateral ventricle 24h after MCAO.
5. The combined therapy group: MCAO rats underwent transplantation of BMSCs and mild hypothermia treatment.

The rats were subcutaneously anesthetized with Zoletil 50 (50 μg/g; Virbac Laboratory, Carros, France). All rats returned to their home cages for recovery, with free access to chow and tap water. Carprofen (5mg/kg, Rimadyl®) was administered for analgesia on the day of surgery and the next 2 days. During housing, animals were monitored twice daily for health status. No adverse events were observed. All animals were euthanized by an overdose of Zoletil 50. No animals died during the experiment period. All sections of this report adhered to the ARRIVE Guidelines for reporting animal research. A completed ARRIVE guidelines checklist is included in S1 Appendix.

### 2.2. Preparation of rat BMSCs

The procedures of isolating and culturing BMSCs were performed as reported previously[14]. The marrow suspension was harvested from both sides of tibia and femur of SD rats via rinsing with 5ml DMEM/F12(GIBCO, USA) medium repeatedly. After centrifugation and filtration, the suspension was mixed with Percoll cell separation solution (1.077 g/ml, GE healthcare, Switzerland) and centrifuged at 1000r/min for 10min, followed by culturing with DMEM/F12 medium containing 15% fetal bovine serum(FBS) and 1% penicillin G and streptomycin (GIBCO, USA) in an incubator under 37°C and 5% CO_2_ volume fraction and saturated humidity. After incubated for 48 h, most non-adherent cells were screened out by replacing the old medium. When the confluence reached 80–90%, primary cells were trypsinized using 0.25% trypsin (GIBCO, USA) and re-plated into two new culture dishes at the ratio of 1:2, which were renamed passage 1 (P1). Phenotypes of BMSCs at P3 were identified for CD 29, CD 105 and CD 45 (eBioscience, USA) by flow cytometry. After identification, BMSCs were incubated with 10 μmol/L BrdU (Sigma Aldrich) for 48 h before transplantation. The next day, BMSCs were harvested and counted at the density of 5×10^4^/μl in PBS for transplantation.

### 2.3. Establishment and filter of MCAO models

The MCAO procedure was a modification of the method reported previously[15]. Briefly, all rats were fasted for about 12h before surgery. After anesthesia, a middle cervical incision was performed and the superficial fascia was dissected. Blunt dissection and careful sharp were performed to identify the right common carotid artery (CCA), internal carotid artery (ICA) and external carotid artery (ECA). A 4–0 monofilament nylon suture with a rounded tip (Beijing Cinontech Co., Ltd, Beijing, China) was inserted into the ICA through a little incision of ECA. The nylon suture was advanced from the ECA to reach the anterior cerebral artery (ACA) through the ICA until a slight resistance was felt. The depth was about 18–20 mm from the bifurcation and the blood flow of the middle cerebral artery (MCA) was blocked. Two hours after MCAO, the nylon suture was slightly withdrawn for reperfusion. During the surgery, the rats were kept at a constant temperature of approximately 37°C using a heating pad and an infrared lamp, and monitored continuously by a thermometer introduced into the rectum. For the sham-surgery group, the right CCA was isolated with no further processing. After the rats recovered from anesthesia, all rats were scored based on the method described previously[16]. This is a five-point scale: 0, no neurological deficit; 1, failure to extend the left forepaw fully; 2, circling to the left; 3, falling to the left; 4, incapable of walking spontaneously and loss of consciousness. The rats with scores of 1 to 3 were chosen to establish MCAO models.

### 2.4. Mild hypothermia treatment

The rectal temperature of rats in the MH group and combination therapy group after MCAO surgery was kept at 30–33°C (corresponding to brain temperature of 32–35°C)[17]. Mild hypothermia began since ischemia onset, and cooling was maintained for 3h by placing the rat’s head on ice bags. Rectal temperature was measured every 15min. After receiving mild hypothermia treatment for 3h, the animals were gradually rewarmed with an infrared lamp and a heating blanket. In normothermia rats, the brain temperature of MCAO rats was maintained at 37.0±0.5°C during the experiment using a heating pad and an infrared lamp.

### 2.5. Lateral ventricle injection

MCAO rats were anesthetized and placed onto a rat brain stereotaxic apparatus 24 h after the MCAO operation. An incision was made on the vertex of the head and bregma was exposed. The bregma was taken as the coordinate origin and the surface projection point of the left lateral ventricle (0.8 mm behind the bregma, and1.5 mm left to sagittal suture) was determined. A burr hole was made using a small dental drill. For the BMSCs group, 20μl of BMSCs (1 x 10^6^) which were incubated with 10 μmol/L BrdU (Sigma Aldrich) for 48 h and suspended in PBS were injected into the left lateral cerebral ventricle at the depth of 4.0 mm lasting for 5min using an automatic micro-injection pump. After pulling out needles slowly, the burr hole was covered with paraffin to prevent cerebrospinal fluid leakage. In the model group, 20μl of sterile PBS was injected into the left lateral cerebral ventricle using the same protocol. All transplantation procedures were performed under aseptic conditions.

### 2.6 BMSCs labeling and cell transplantation

Before transplantation, BMSCs were incubated with 10 μmol/L BrdU (Sigma Aldrich) for 48 h. Twenty-four hours after MCAO, approximately 1 × 10^6^ BMSCs in 1 ml PBS were injected into the rat via the tail vein.

### 2.7. Evaluation of neurological function

All rats were examined in the neurological function test at five respective time- points: before MCAO surgery, before transplantation, at day 1, day 5 and day 10 after transplantation, by two investigators who were blinded to this study using mNSS[18], including motor test, sensory test, beam balance test and reflexes absence and abnormal movement test.

### 2.8. TTC staining

Six rats selected from each sub-group randomly were anesthetized after neurological function assessment at day 1, day 5 and day 10 after transplantation. After perfused with cold normal saline intracardially, the rat brain was harvested carefully and frozen at -20°C for 30min. The brain was cut into 2-mm-thick coronal slices with a rat brain slice mold. The brain slices were stained with 1% TTC solution (Sigma, USA) in PBS at 37°C for 30min in the dark, and then fixed in 4% paraformaldehyde solution for 24h. After staining, the normal tissues were stained red while the infarct zone was stained white. Slices were captured and measured using Image-J analysis software (National Institutes of Health, USA) to calculate the percentage of the infarct area.

### 2.9. Immunofluorescence staining

Immunofluorescence staining was performed to observe BMSCs' homing, and to detect the expression and variation tendency of GFAP and VEGF in the ischemia area. After harvesting brain tissues, sequential coronal slices (6μm thick for each slice) were cut using a frozen slicer (Leica, Germany), where the temperature was kept at -20°C, followed by adhering the brain slices to the slides coated by poly-lysine. For BMSCs homing assay, the sections were first pretreated to denature DNA as follows: after immersion in 50% formamide/2× saline-sodium citrate buffer (SSC) at 65°C for 2 h, the sections were washed in PBS for 10 min, incubated in 2 N HCl at 37°C for 30 min, and washed with 0.1 M boric acid (pH 8.5) for 10 min. The sections were then incubated with a mouse anti-BrdU (1:100; Sigma Aldrich) at 4°C overnight, washed in PBS, and then incubated with secondary antibodies of Alexa Fluor 488-conjugated goat anti-mouse IgG (Thermo Fisher Scientific Inc., MA, USA) for 1 h at 37°C. Counterstaining was done with 4′,6-diamidino-2-phenylindole (DAPI; Zhongshan Golden Bridge Biotechnology, Beijing, China). For determination of the GFAP and VEGF expression, the slices were incubated respectively with individual primary anti-GFAP polyclonal antibody (1:200, Abcam, UK) and anti-VEGF (1:100, Abcam, UK) polyclonal antibody overnight at 4°C. After rinsing, slices were incubated with Alexa Fluor 488-conjugated donkey anti-rabbit antibody (1:200; Life Technology, USA) for 1 hour at 37°C, followed by nuclear staining with PI dye. The same procedures described above except that the primary antibodies were omitted were performed for the negative control slices. All slices were visualized using an inverted fluorescence microscope (Olympus, Japan) at 40X magnification following the standard protocol. At least 5 random images were chosen from each slice or group at each time point.

### 2.10. Immunohistochemistry staining

Paraffin-embedded coronal sections (10 μm thick) were used for the neuropathological analyses. The sections were pre-treated in microwave, and then incubated with 2% hydrogen peroxide in PBS for 15 min at RT to quench endogenous peroxidase activity followed by 10-minute incubation with goat serum to block non-specific binding. Then sections were incubated with mouse monoclonal anti-RECA-1 (1:500; Abcam, Shanghai, China) overnight at 4°C. Immunoreactivity was detected using the ABC protocol followed by staining with diaminobenzidine (Boster, Wuhan, China). All the sections were briefly counterstained to show cell nuclei with hematoxylin, and examined under a light microscope. The microvessels (RECA-1-positive structures) were examined within the temporal cortex under × 100 magnification.

### 2.11. Western blot analysis

Procedures were performed according to the methods described before[17]. Six rats chosen from each sub-group randomly were anesthetized and killed at day 1, day 5 and day 10 after transplantation. The brain was removed carefully, and the right cortical tissues of MCA regions were collected. They were frozen in liquid nitrogen immediately, and then stored in a -80°C fridge until use. The total protein was extracted and its concentration was determined using a BCA protein assay kit (Pierce Biotechnology Inc., Rockford, USA). The total protein was subjected to protein electrophoresis and transferred to nitrocellulose membranes (BIO-RAD, USA). The membrane was placed into 5% skimed milk for 1h, and incubated with primary antibodies anti-GFAP (1:1000, abcam, UK) and anti-VEGF (1:1000, abcam, UK) overnight at 4°C. The membrane was incubated with appropriate secondary antibodies conjugated with horseradish peroxidase (Santa Cruz Biotechnology, USA) and then the membranes were exposed to X-ray films. Normalization of results was ensured by running parallel western blot, with β-actin antibody as the internal control. The expression of GFAP and VEGF was then normalized to β-actin.

### 2.12. Statistical analysis

The mNSS scores and the percentage of cerebral infarction areas was expressed as mean ± standard deviation (mean±SD) and analyzed by GraphPad Prism 5 statistical software (GraphPad Software, USA). The intergroup difference was analyzed using one-way analysis of variance (ANOVA). p<0.05 indicated significant difference. To minimize the variation in fluorescence intensities between images, absolute number of pixels only was compared between sets of images, which were taken on the same microscope under identical conditions and were stained in the same experiment with the same pool of antibodies.

## 3. Results

### 3.1. The combination therapy promoted the neurofunctional improvement

**Fig 1:**
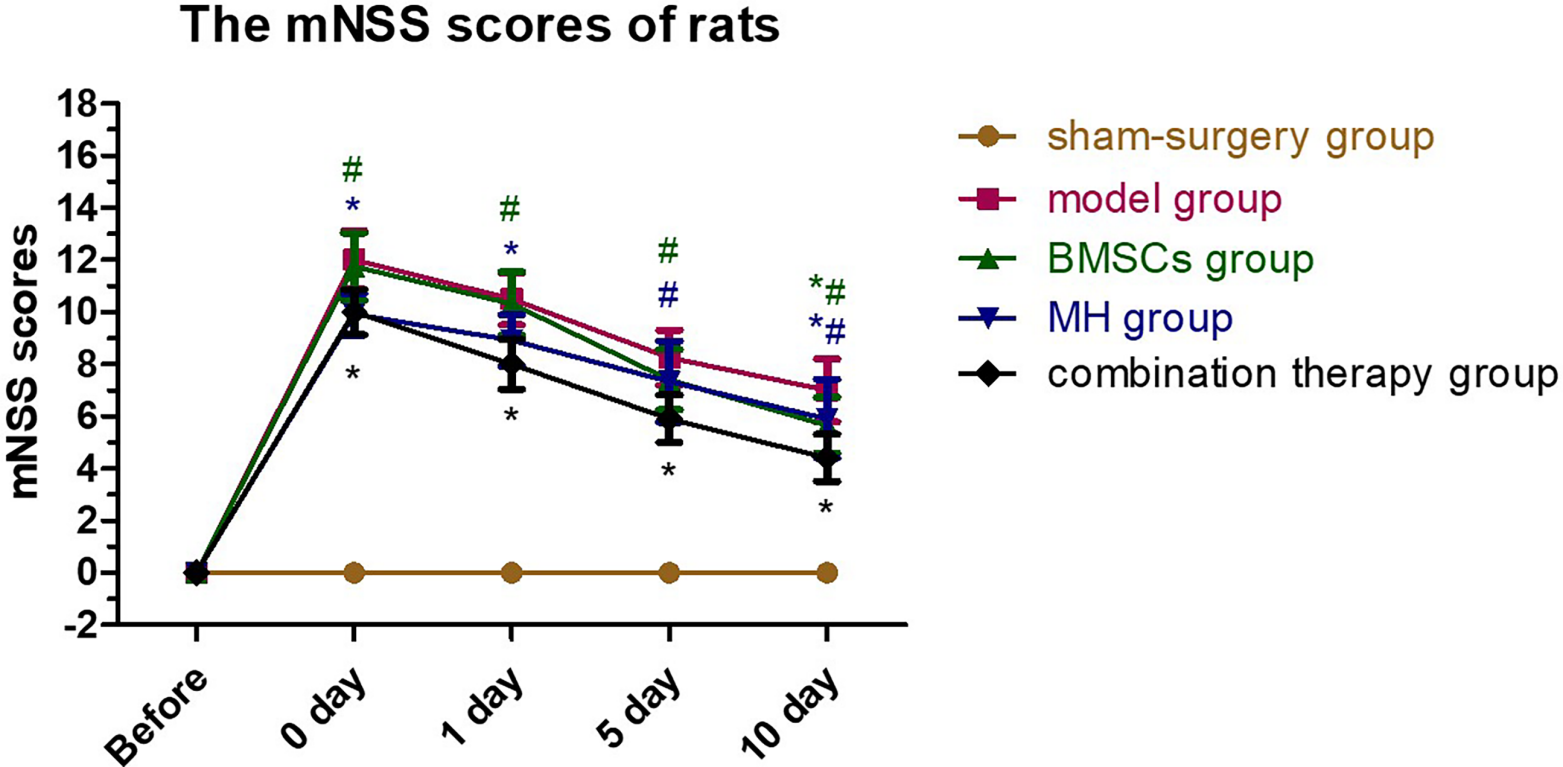
Variation tendency of rats mNSS scores. Neurological functional tests were performed in each group at each time point (n = 6), respectively. The rats mNSS scores in the combination therapy group decreased more obviously than that in the BMSCs group. Values are expressed as mean± SE. *P < 0.05, compared to control; ^#^P<0.05, compared with the combined therapy group.

After right MCAO operation, the neurological deficits of rats varied in the groups. The rats in the sham-surgery group had no neurological deficits, and the rats in the other four groups had the highest mNSS scores before transplantation. For the combination therapy group, the mNSS scores of the rats decreased significantly before transplantation, at day 1, day 5 and day 10 after transplantation (P<0.01) compared with the BMSCs group; compared with the MH group, the mNSS scores decreased significantly at day 5 and day 10 after transplantation (P<0.01), but the reduction was not obvious before transplantation and at day 1 after transplantation (P>0.05). The mNSS scores of each group are shown in Table 1, and the variation tendency is shown in Fig 1.

**Table 1:** The mNSS scores of rats.

Group	sham-surgery	model	BMSCs	MH	combination therapy
Before MCAO	0.00±0.00	0.00±0.00	0.00±0.00	0.00±0.00	0.00±0.00
Before transplantation	0.00±0.00	12.00±1.08	11.75±1.23^#^	9.92±0.76*	10.00±0.82*
1d after transplantation	0.00±0.00	10.50±0.96	10.33±1.18^#^	8.92±0.95*	8.00±0.91*
5d after transplantation	0.00±0.00	8.25±1.01	7.42±1.11^#^	7.33±1.49^#^	5.92±0.86*
10d after transplantation	0.00±0.00	7.00±1.15	5.67±1.03*^#^	5.92±1.44*^#^	4.42±0.86*

Neurological functional tests were performed in each group at each time point (n = 6), respectively. The rats mNSS scores in the combination therapy group decreased more obviously than that in the BMSCs group and MH group.

*P<0.05 compared with the model group

^#^P<0.05, compared with the combination therapy group. Datas were shown as mean±SD

### 3.2. The combination therapy decreased the infarction size

**Fig 2:**
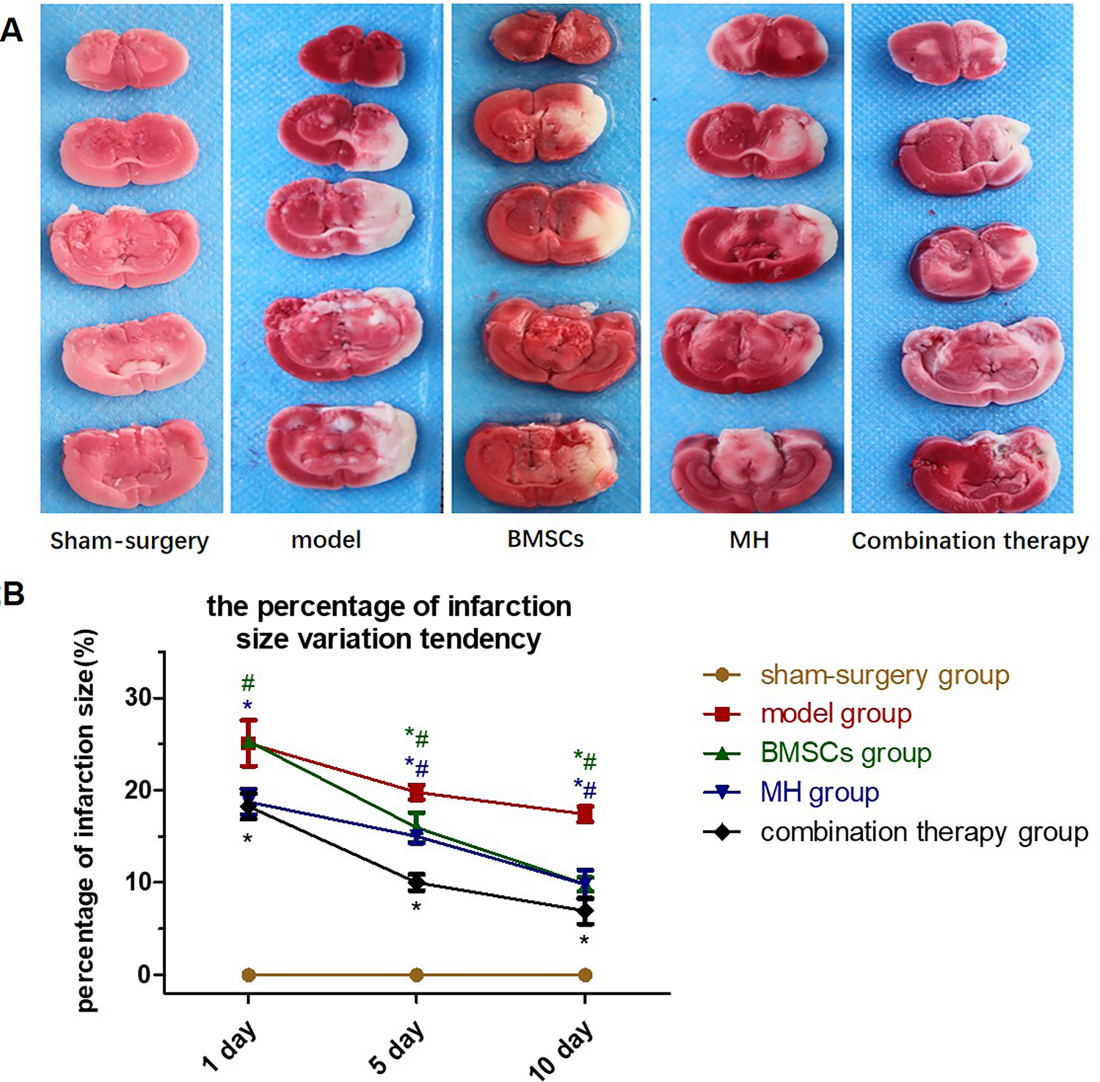
The effects of BMSCs transplantation combined with therapeutic mild hypothermia on the infarct area which were confirmed by TTC staining at day 1, day 5 and day 10 after transplantation. Normal tissues were stained red, and the infarcted tissues were stained white. A. TTC staining showed that the infarction area was located at the territory of MCA in the right hemisphere with stable staining position; B. The percentage infarction size was calculated in each group at each time point (n = 6), respectively. Values are expressed as mean± SE. *P < 0.05, compared to control; ^#^P<0.05, compared with the combined therapy group.

TTC staining showed that the infarction area was located at the territory of MCA in the right hemisphere with stable staining position (Fig 2A). Normal tissues were red after staining, and infarcted tissues were white after staining. For the combination therapy group, the percentage of the infarct area was lower at all time-points (P<0.01) compared with BMSCs group; compared with MH group, the percentage of the infarct area decreased at day 5 and day 10 after transplantation (P<0.01), while no statistical diminution was found at day 1 after transplantation (P>0.05). The percentage of the infarct area in each group is shown in Table 2, and the variation tendency is shown in Fig 2B.

**Table 2:** The percentage of infarction size of rats.

Group	sham-surgery	model	BMSCs	MH	combination therapy
1d after transplantation	0.00±0.00	20.10±2.27	25.29±0.52^#^	18.75±1.25*	18.25±1.25*
5d after transplantation	0.00±0.00	19.78±0.72	15.96±1.45*^#^	14.99±0.65*^#^	10.01±0.81*
10d after transplantation	0.00±0.00	17.41±0.75	9.82±0.69*^#^	9.80±1.42*^#^	6.92±1.28*

The percentage infarction size was calculated in each group at each time point (n = 6), respectively.

*P<0.05 compared with the model group

^#^P<0.05, compared with the combination therapy group.Datas were shown as mean±SD (%)

### 3.3. Changes in GFAP expression in different groups

**Fig 3:**
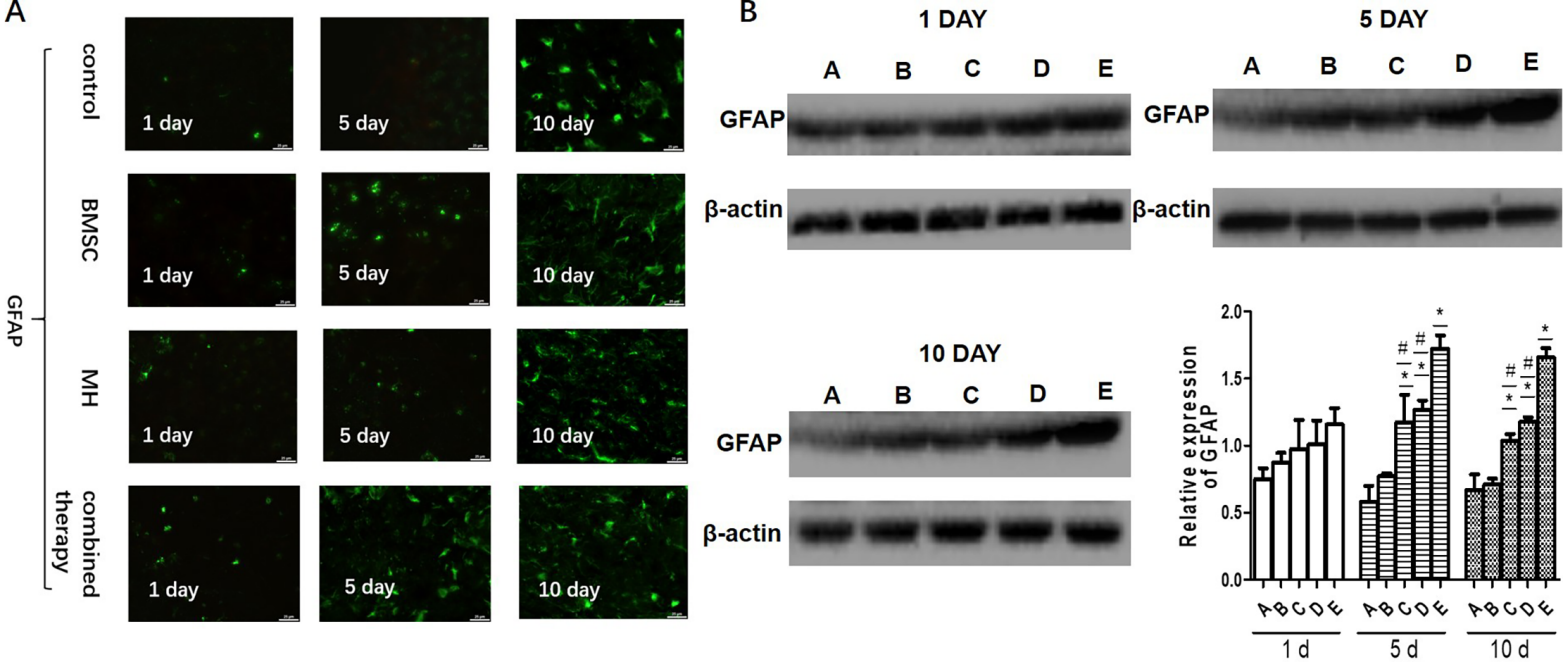
The combination therapy increased the expression of GFAP in ischemic regions A. The expression of GFAP was determined by immunofluorescence. B. The expression of GFAP was measured by western blot. (A: sham-surgery group; B:model group; C:BMSCs group; D:MH group; E:combination therapy group.) Values are expressed as mean± SE. n = 3. *P < 0.05, compared to control; ^#^P<0.05, compared with the combined therapy group.

The GFAP positive cells were observed in the BMSCs group and combination therapy group at each time point after transplantation under the inverted fluorescence microscope. Compared with the BMSCs group and MH group, the number of GFAP positive cells and fluorescence density of protein GFAP in the combination therapy group showed no significant changes at day 1, but increased obviously at day 5 and day 10 after transplantation. The results of immunofluorescence staining are shown in Fig 3A.

Western blotting analyses showed that the expression of GFAP increased at day 5 and day 10 after transplantation, respectively, in the brain of cerebral ischemia rats treated with BMSCs or mild hypothermia alone. For the combination therapy group, the expression of GFAP increased significantly compared with the BMSCs group and MH group at day 5 and day 10 after transplantation, respectively (P<0.01) (Fig 3B). It was suggested that the combination therapy increased the expression of GFAP in the ischemic brain compared with BMSC group and MH group.

### 3.4 The combination therapy enhanced BMSCs homing into the ischemic brain

**Fig 4:**
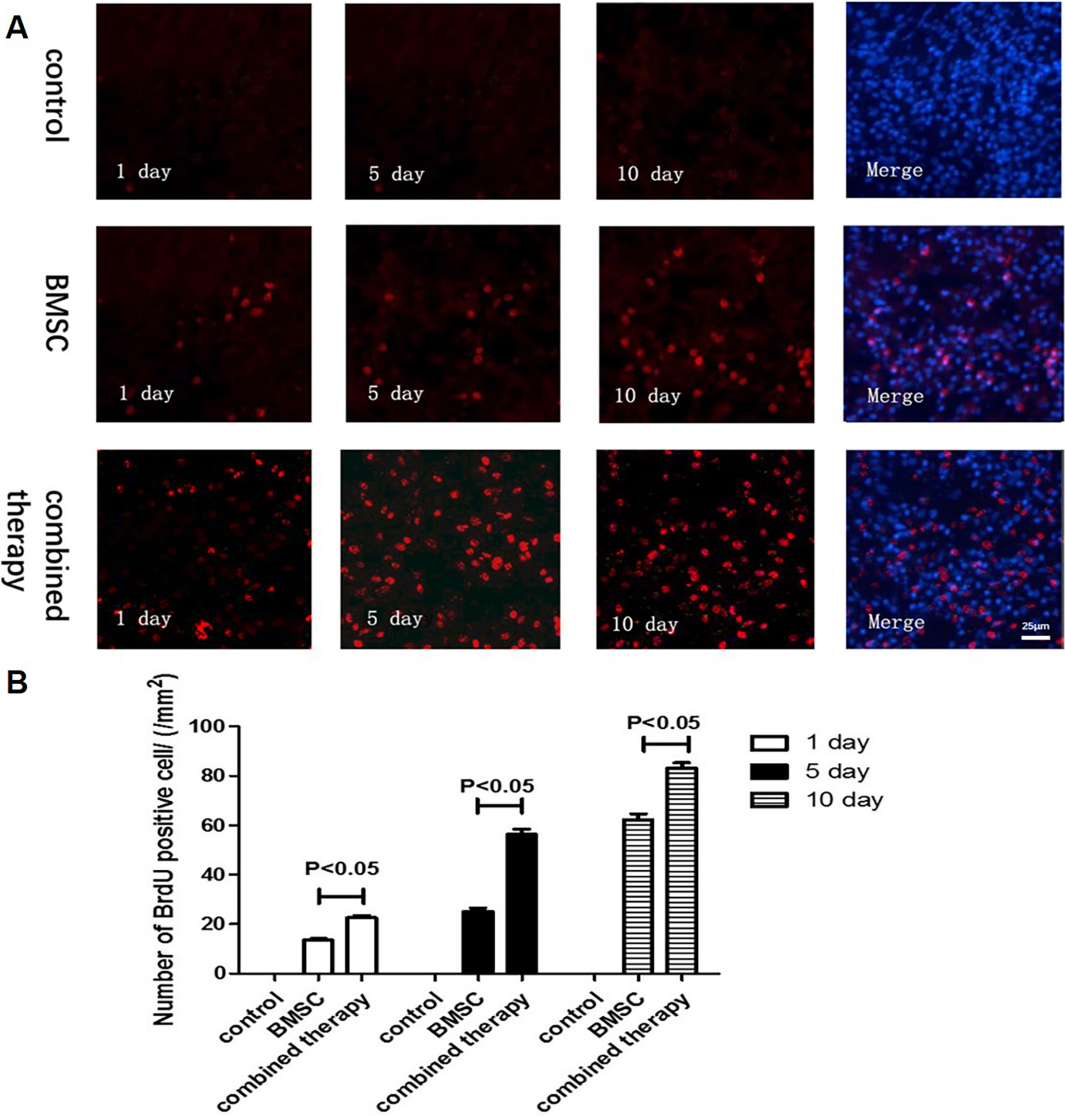
The combination therapy promoted BMSCs homing into the ischemic brain. BMSCs labeled by BrdU (red) and nuclei labeled with DAPI (blue) were observed at the lesion after MCAO. A. The homing ability was evaluated at day 1, day 5 and day 10 after MCAO. B. Quantification of migrated BMSCs. Values are expressed as mean± SE. n = 6.

To detect the homing efficiency of injected BMSCs, the number of BrdU-positive BMSCs was counted in the ischemic regions. In the penumbra cortex, the combination therapy group increased BMSCs homing efficiency compared with the BMSCs group on day 5 and day 10 after MCAO (Fig 4A and 4B). It was indicated that the combination therapy enhanced BMSCs homing into the ischemic brain.

### 3.5 The combination therapy increased angiogenesis in ischemic regions

**Fig 5:**
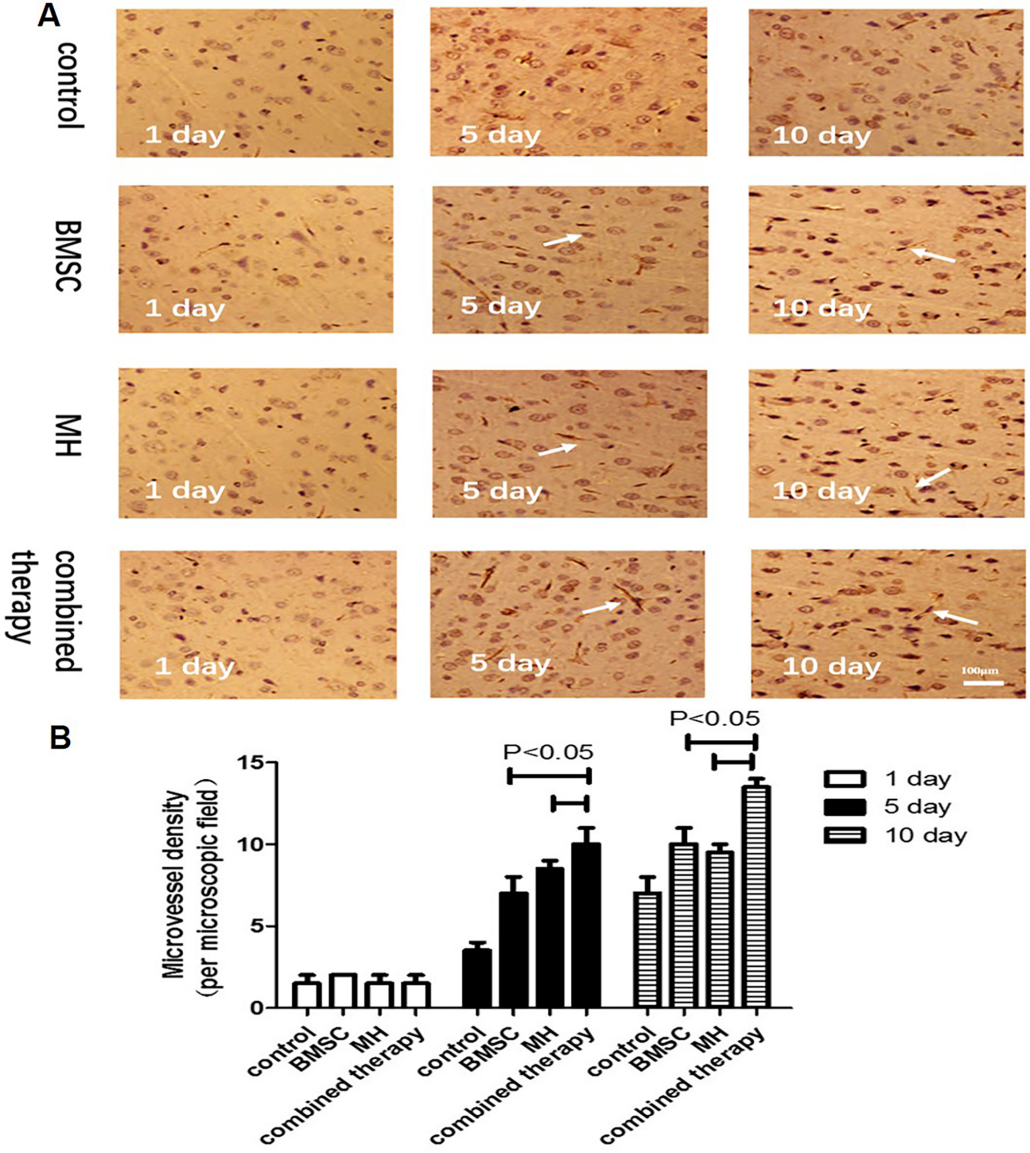
The combination therapy increased the expression of VEGF in ischemic regions. A. The expression of VEGF was determined by immunofluorescence. B. The expression of VEGF was measured by western blot. (A: sham-surgery group; B:model group; C:BMSCs group; D:MH group; E:combination therapy group.) Values are expressed as mean± SE. n = 3. *P < 0.05, compared to control; ^#^P<0.05, compared with the combined therapy group.

**Fig 6:**
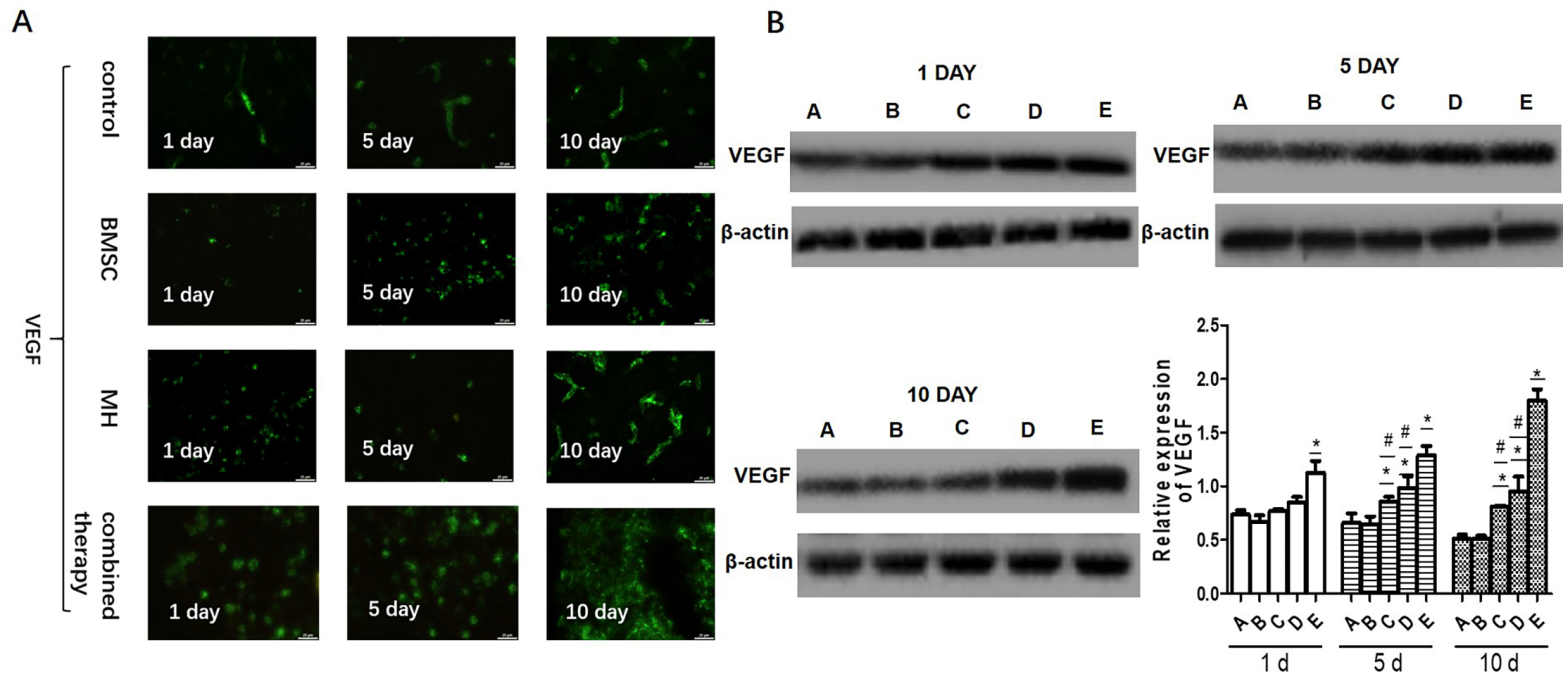
The combination therapy increased angiogenesis in ischemic regions. Microscopic images of the temporal cortex in coronal sections stained with anti-RECA-1 antibody. The arrows indicate RECA-1 positive microvessels. A. The microvessels (RECA-1 positive structures) were examined within the temporal cortex under × 100 magnification. B. Quantification of microvessels is expressed as microvessel density (number of stained vessels per microscopic field). Data are expressed as mean± S.E. n = 3.

VEGF is an important signaling protein involved in the angiogenesis (the growth of blood vessels from pre-existing vasculature). To determine the expression of VEGF in the ischemic brain of various group, the immunofluorescence staining and Western blotting were used.The results showed that the combination therapy increased the expression of VEGF in the ischemic brain compared with BMSC group and MH group on day 5 and day 10 after MCAO(Fig 5A and 5B). The previous study showed that VEGF promoted angiogenesis in the brain, thereby contributing to the protection of brain cells from ischemic injury. To further verify whether the brain protection was associated with any increased angiogenesis in the brain after BMMSC transplantation. Immunohistochemistry using a specific antibody against RECA-1, a specific cell surface marker of rat vascular endothelial cells, showed the density of microvessels in the temporal cortex on day 1, day 5 and day 10 after MCAO. There was no significant difference in the RECA-1 positive cells among different groups on day 1 after MCAO. However, compared with the MCAO group, BMSCs transplantation and MH treatment significantly increased RECA-1 positive cell on day 5 and day 10 after MCAO, respectively. Moreover, MCAO combined with transplantation of BMSCs and MH treatment increased RECA-1 positive cells compared to the MH group and BMSCs transplantation group on day 5 and day 10 after MCAO (Fig 6A and 6B). It was indicated that the combination therapy increased angiogenesis in ischemic regions.

## 4. Discussion

According to our results, the mNSS scores and the percentage of the infarct area of the rats in the combination therapy group was significantly lower than that in the BMSCs group and MH group, and the homing and angiogenesis were significantly increased in the combined therapy group than in the MH group and BMSCs transplantation group on day 5 and day 10 after MCAO. Besides, the rats in the combination therapy group had higher expression levels of GFAP and VEGF on day 5 and day 10 after transplantation. The experimental results seemed to verify the hypothesis that the combination of BMSCs transplantation and mild hypothermia treatment contributes to better therapeutic effects than a single treatment.

Numerous studies have shown that BMSCs transplantation and therapeutic mild hypothermia significantly improve neurological functional outcomes and decrease the infarct area caused by MCAO, respectively[7, 14, 18, 19]. However, there are few reports on the combination of the two different neuroprotective strategies for the treatment of cerebral ischemia. Coupled with the other manuscript demonstrating its neuroprotective role on ischemic stroke[20–23], it is strongly suggested that combination of BMSCs transplantation and MH can significantly reduce infarction size and improve functional recovery by promoting neurogenesis and angiogenesis, which may be a beneficial treatment for cerebral ischemia.

In the animal model of cerebral ischemia, BMSCs transplantation could improve neurological functional outcomes and decrease the infarct area via differentiation, replacement and neural circuit reconstruction[24], enhancing angiogenesis[25], facilitating neurotrophic factor secretion[26], and reducing cell apoptosis[5, 27]. Previous studies showed that BMSCs transplantation after lacunar stroke induced by injecting Ouabain stereotaxically could enhance neurogenesis in the sub-ventricular zone (SVZ), express astrocytes (AS) phenotype GFAP, and enhance neurological functional recovery[6]. Astrocytes provide adequate nutrients to maintain the metabolism of nerve cells and regulate synaptic activity to maintain the balance between synapses. After cerebral ischemia, endogenous AS could be activated and proliferated to protect nerve cells from oxidative stress injury. Furthermore, Deng found that BMSCs transplantation was an effective treatment to promote the repair of damaged brain tissues, reduce neuronal apoptosis and promote the proliferation of neurons by increasing the expression of VEGF[28]. Angiogenesis within the infarction zone is thought to play a vital role in mediating survival and regeneration of neurons after stroke. VEGF promotes angiogenesis in the brain, thereby contributing to the protection of brain cells from ischemic injury. Therefore, a specific cell surface marker of rat vascular endothelial cells, RECA-1, was used to determine the density of microvessels in the temporal cortex on day 1, day 5 and day 10 after MCAO. In our study, the combined therapy increased the microvessel density on day 10 after MCAO compared with the MH group and BMSC transplantation group, suggesting that the combination therapy increased angiogenesis in ischemic regions. What’s more, compared with the model group, the mNSS scores in the BMSCs group decreased at day 10 after transplantation and the percentage of the infarct area decreased at day 5 and day 10 after transplantation, indicating that BMSCs transplantation may treat cerebral ischemia through increasing GFAP expression and promoting angiogenesis. Compared with the BMSCs group, the number of GFAP and VEGF^+^ double positive cells and fluorescence density of protein GFAP and VEGF in the combination therapy group showed no significant changes at day 1 after transplantation, but increased obviously at day 5 and day 10 after transplantation. Western blotting results showed that the expression levels of GFAP and VEGF in the combination therapy group increased significantly compared with the BMSCs group at all time-points after transplantation. Also, the mNSS scores and the percentage of the infarct area in the combination therapy group were lower at all time-points compared with BMSCs group. Therefore, it was concluded that the combination therapy remarkably improved functional outcomes and reduced the infarction area compared with a single BMSCs treatment.

Mild hypothermia is an established treatment in the laboratory, which has remarkable effects on brain injury. Reducing the intracranial temperature mildly could slow down blood flow, reduce the consumption of oxygen and glucose by promoting the dissociation curve of oxygenated hemoglobin shift to the left, and lower cerebral metabolism[29]. Therefore, it could prevent accumulation of lactic acid, and maintain stability of the internal environment PH. Xie YC reported that mild hypothermia treatment was associated with expanding the microvessel diameter, increasing the number of vascular branch points and improving the vessel surface area within the ischemic hemisphere[30]. In our study, MH treatment significantly increased microvessel density on day 10 after MACO. When MH treatment was combined with BMSCs transplantation, the microvessel density was further increased compared with MH treatment alone, suggesting that the combined therapy showed a promising application prospect. In addition, mild hypothermia could significantly reduce the expression of FAS and alter the extrinsic Fas/FasL apoptotic pathway[31]. By comparing the data in the model group and MH group, it was found that the expression levels of GFAP in the MH group were higher from day 1 after transplantation and the expression levels of VEGF in the MH group increased with the observation time extended. Furthermore, the MH group had significantly decreased mNSS scores and percentage of the infarct area compared with the model group before transplantation and at day 1 after transplantation, while there were no significant changes in the BMSCs group. Also, the immunofluorescence staining results showed that the number of cells in the MH group was higher than that in the BMSCs group at day 1 after transplantation. The above experimental data showed that mild hypothermia treatment played a neuroprotective role in the acute and subacute stage of ischemia stroke and a longer period (more than 5 days) was required for BMSCs treatment to perform the therapeutic effect. Through further comparison between the combination therapy group and MH group, it was found that the expression levels of GFAP and VEGF increased, and the mNSS scores and the percentage of the infarct area decreased at early stage of cerebral ischemia, indicating that the combination therapy had a better performance than MH alone.

BMSCs not only increased angiogenesis in ischemic regions, but also promoted VEGF and GFAP expression, playing a protective role in the brain. Previous study showed that mild hypothermia treatment could slow down the brain blood flow, reduce brain metabolism, interrupt the accumulation of acid metabolites[32], and attenuate the process of cell apoptosis[33] in the acute and subacute stage of ischemic stroke. In addition, through reducing acidic metabolites accumulation in the ischemic area and preventing generation of excitotoxic neurotransmitters, mild hypothermia improved the focal environment after cerebral ischemia to provide favorable internal environment for BMSCs to perform the protective role in the treatment of cerebral ischemia. In our study, the combined therapy promoted BMSCs homing and angiogenesis, and increased the expression of GFAP and VEGF compared to the MH group and BMSC group. Our study showed that the combination of BMSCs transplantation and mild hypothermia had a better therapeutic effect than a single treatment, which provides a new option for the treatment of cerebral ischemia.

## 5. Conclusion

In conclusion, BMSCs transplantation combined with MH can significantly reduce infarction size and improve functional recovery by promoting homing and angiogenesis, which may be a promising option for the treatment of cerebral ischemia in the future.

## Supporting information

S1 Appendix Checklist. (JPG)

S2 Appendix Abbreviations. (DOCX)
