# Supplementary figures and images for: Bone mesenchymal stem cells transplantation combined with mild hypothermia improves the prognosis of cerebral ischemia in rats
Source: PLoS One. 2018 Aug 1;13(8):e0197405. doi: 10.1371/journal.pone.0197405 (PMC6070180; doi:10.1371/journal.pone.0197405)

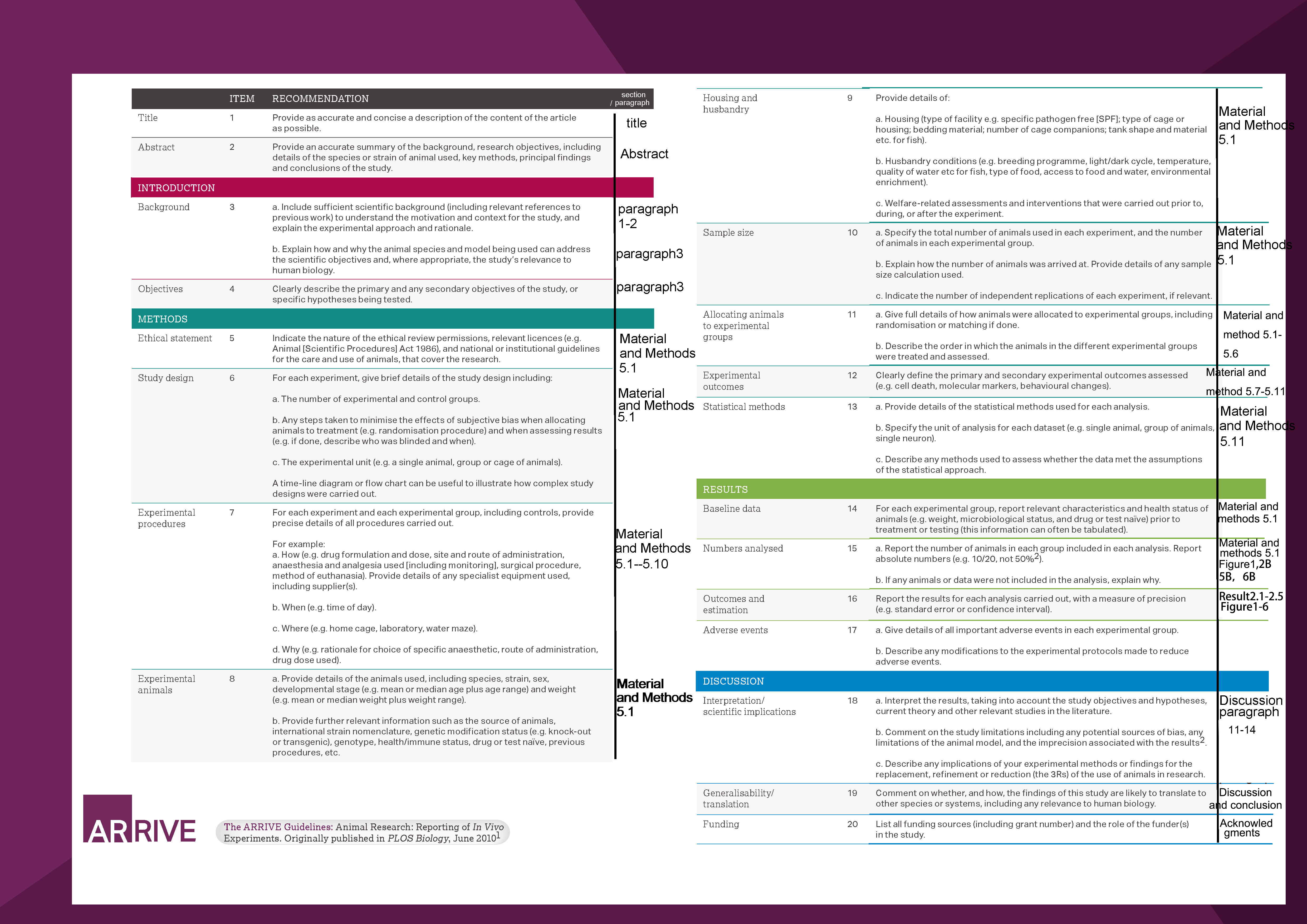

Supplement: S1 Appendix — (JPG) [file pone.0197405.s001.jpg]
