# Supplementary material for: Bone mesenchymal stem cells transplantation combined with mild hypothermia improves the prognosis of cerebral ischemia in rats
Source: PLoS One. 2018 Aug 1;13(8):e0197405. doi: 10.1371/journal.pone.0197405 (PMC6070180; doi:10.1371/journal.pone.0197405)
Supplement: S2 Appendix — (DOCX) [file pone.0197405.s002.docx]

**Abbreviations:**

ACA: anterior cerebral artery;

AS: astrocyte;

ANOVA: one-way analysis of variance

BMSCs: bone marrow mesenchymal stem cells;

CNS: central nervous system;

CCA: common carotid artery;

DAPI: 4,6-diamidino-2-phenylindole;

ECA: external carotid artery;

FBS: fetal bovine serum;

FAS: factor associated suicide;

FASL: factor associated suicide ligand;

GFAP: glial fibrillary acidic protein;

ICA: internal carotid artery;

ICH: intracranial hemorrhage;

MH: mild hypothermia;

MCAO: middle cerebral artery occlusion;

MCA: middle cerebral artery;

mNSS: modified Neurological Severity Score;

MSCs: mesenchymal stem cells，

PBS: phosphate buffer solution;

rt-PA: recombinant tissue plasminogen activator;

RECA-1:rat vascular endothelial cells

SVZ: sub-ventricular zone;

SD: Sprague-Dawley;

SPF: specific-pathogen-free level

SSC: saline-sodium citrate buffer

TTC: 2,3,5-triphenyl tetrazolium chloride;

VEGF: vascular endothelial growth factor;
